# Detecting, Characterizing, and Mitigating Implicit and Explicit Racial Biases in Health Care Datasets With Subgroup Learnability: Algorithm Development and Validation Study

**DOI:** 10.2196/71757

**Published:** 2025-09-04

**Authors:** Faris Gulamali, Ashwin Shreekant Sawant, Lora Liharska, Carol Horowitz, Lili Chan, Ira Hofer, Karandeep Singh, Lynne Richardson, Emmanuel Mensah, Alexander Charney, David Reich, Jianying Hu, Girish Nadkarni

**Affiliations:** 1 Icahn School of Medicine at Mount Sinai, 1468 Madison Avenue, New York, NY, 10029, United States, 1 2122416500; 2 University of California, San Diego, San Diego, CA, United States; 3 Christiana Care Health System, Wilmington, DE, United States; 4 IBM Research, Yorktown Heights, NY, United States

**Keywords:** machine learning, data-centric artificial intelligence, bias, fairness

## Abstract

**Background:**

The growing adoption of diagnostic and prognostic algorithms in health care has led to concerns about the perpetuation of algorithmic bias against disadvantaged groups of individuals. Deep learning methods to detect and mitigate bias have revolved around modifying models, optimization strategies, and threshold calibration with varying levels of success and tradeoffs. However, there have been limited substantive efforts to address bias at the level of the data used to generate algorithms in health care datasets.

**Objective:**

The aim of this study is to create a simple metric (AEquity) that uses a learning curve approximation to distinguish and mitigate bias via guided dataset collection or relabeling.

**Methods:**

We demonstrate this metric in 2 well-known examples, chest X-rays and health care cost utilization, and detect novel biases in the National Health and Nutrition Examination Survey.

**Results:**

We demonstrated that using AEquity to guide data-centric collection for each diagnostic finding in the chest radiograph dataset decreased bias by between 29% and 96.5% when measured by differences in area under the curve. Next, we wanted to examine (1) whether AEquity worked on intersectional populations and (2) if AEquity is invariant to different types of fairness metrics, not just area under the curve. Subsequently, we examined the effect of AEquity on mitigating bias when measured by false negative rate, precision, and false discovery rate for Black patients on Medicaid. When we examined Black patients on Medicaid, at the intersection of race and socioeconomic status, we found that AEquity-based interventions reduced bias across a number of different fairness metrics including overall false negative rate by 33.3% (bias reduction absolute=1.88×10^−1^, 95% CI 1.4×10^−1^ to 2.5×10^−1^; bias reduction of 33.3%, 95% CI 26.6%‐40%; precision bias by 7.50×10^−2^, 95% CI 7.48×10^−2^ to 7.51×10^−2^; bias reduction of 94.6%, 95% CI 94.5%‐94.7%; false discovery rate by 94.5%; absolute bias reduction=3.50×10^−2^, 95% CI 3.49×10^−2^ to 3.50×10^−2^). Similarly, AEquity-guided data collection demonstrated bias reduction of up to 80% on mortality prediction with the National Health and Nutrition Examination Survey (bias reduction absolute=0.08, 95% CI 0.07-0.09). Then, we wanted to compare AEquity to state-of-the-art data-guided debiasing measures such as balanced empirical risk minimization and calibration. Consequently, we benchmarked against balanced empirical risk minimization and calibration and showed that AEquity-guided data collection outperforms both standard approaches. Moreover, we demonstrated that AEquity works on fully connected networks; convolutional neural networks such as ResNet-50; transformer architectures such as VIT-B-16, a vision transformer with 86 million parameters; and nonparametric methods such as Light Gradient-Boosting Machine.

**Conclusions:**

In short, we demonstrated that AEquity is a robust tool by applying it to different datasets, algorithms, and intersectional analyses and measuring its effectiveness with respect to a range of traditional fairness metrics.

## Introduction

The adoption of diagnostic and prognostic algorithms in health care has led to concerns about the perpetuation of bias against underrepresented groups. These biases exist even in algorithms trained using diverse populations, because longstanding societal biases and disparities are reflected in the data used to develop algorithms [1].

Disparities in health care by race, socioeconomic status, and gender have been documented for decades [2]. As a result, data generated from health care datasets, which contain these disparities, can produce models that reproduce health inequity [3,4]. In machine learning for population health, these disparities can be broadly categorized as based on the prediction of outcomes, identification of causal factors, design of interventions, and resource allocation [5]. Bias is often defined as the violation of group fairness, in this case the expectation that machine learning models should perform similarly for different demographic groups [6]. Although this metric can be suitable for the prediction of outcomes, it does not necessarily apply to resource allocation, identification of causal factors, or intervention design [7,8]. It has been shown that the use of models that predict health care costs results in underallocation of resources to Black patients, because the dataset reflects the fact that Black patients have been historically underserved by the health care system [8].

Attempts to mitigate bias have focused on fairness metric–based algorithm optimization after training on the data [2,6,9]. There is increasing recognition of the need to intervene early in the algorithm development pipeline, when data are collected and curated or when outcomes are chosen [2,6,9]. Data-centric artificial intelligence is a rapidly growing field, both in the health care space and out of it [10,11]. Several data-based approaches have been generated recently including data generation [12]. Other examples such as importance weighting, fairness batching, fair active learning, and slice tuning are all examples of modifying the dataset before training the model [13-15]. Although these approaches have been met with moderate success, these methods are not as practical in the context of health care research where (1) selection of unbiased outcomes remains an important issue, (2) the collection of raw data is guided via empirical demographic factors, and (3) the methods used are often complex. Although some data collection approaches have been discussed, the state-of-the-art method remains balanced empirical risk minimization [16].

In this paper, we explore how the investigation of differences in data distribution between subgroups in a population can help diagnose and mitigate different manifestations of bias. Based on whether the populations with a given outcome are similar or not, we can recommend either prioritizing the collection of additional data from the disadvantaged subgroup or choosing a different outcome measure.

We call our method AEquity, based on its reliance on an autoencoder architecture and its aim of promoting equity. Although current methods primarily focus on reweighting existing data, batching data in equitably distributed groups, or robustly optimizing individual groups independently, AEquity is novel in that it functions at small sample sizes and can identify issues with both the independent variables (the dataset) and the dependent variables (the outcomes or labels). Finally, AEquity can function with nearly any machine learning model, including large multimillion-parameter models such as vision transformers and those that are nondifferentiable like gradient boosted trees.

An autoencoder is a type of machine learning model that only takes in the input data and generates a compressed representation of the data. We demonstrate its application to detect and mitigate bias in two scenarios wherein health care algorithms have been shown to be biased: (1) an algorithm used to predict health care costs, which exhibits bias against Black patients [8] (health care costs scenario), and (2) a standard computer vision algorithm applied to chest radiographs results in selective underdiagnosis in people who are Black, non-White Hispanic/Latino, or on Medicaid [17] (chest radiographs scenario). Finally, we use this method to detect, characterize, and mitigate a previously unreported, undetected bias in prediction of mortality with the National Health and Nutrition Examination Survey dataset.

In short, the goal of this paper is to demonstrate the validity of AEquity as a data-centric tool across multiple bias types, data modalities, and model architectures.

## Methods

A classification task is a common machine learning task where an algorithm creates a mapping from an input dataset to a set of labels. We followed the framework provided by Corbett-Davies et al [18]. Consider a classification task with examples x drawn from $X\subset R^{n}$. Suppose each x has a binary outcome Y(x), called the label, and a predicted outcome $\hat{Y}\left(x\right)$. Suppose also that X can be partitioned into two mutually exclusive sets $X_{A}$ and $X_{B}$ on the basis of a sensitive characteristic, like age or sex. Let Q be a performance metric (such as area under the receiver operating characteristic curve [AUROC] or false negative rate) that depends on $X,Y,\hat{Y}$ and that $\left|Q\left(X_{A},\;Y,\;\hat{Y}\right)-Q\left(X_{B},\;Y,\;\hat{Y}\right)\right|\;>0$.

We define bias to be the case wherein the prediction is not independent of the example’s sensitive characteristic:


$$\hat{Y}\left(x\right)\;\text{⧸}\;⊥\;\;\;⊥\;\;x\in X_{A}\;\vee \;x\in X_{B}$$

In other words, when a model makes a prediction, a characteristic like age or gender can affect the model’s performance on a given label; for example, if a model is predicting pneumonia, a biased model is one that produces different predictions for patients of different races despite otherwise similar patient characteristics.

This can be further characterized into two separate kinds of bias. The first is performance-affecting bias, where group characteristics are directly linked to patient outcomes.


$$\left|Q\left(X_{A},\;Y,\;\hat{Y}\right)-Q\left(X_{B},\;Y,\;\hat{Y}\right)\right|\;>0$$

for some performance metric of interest Q.

An example would be a diagnostic classifier for chest radiographs that has a higher underdiagnosis rate (false negative rate) for patients who are Black compared to those that are White [17].

However, another type of bias also exists. It has been known that the label chosen for machine learning models can lead to bias insofar as it does not represent the health care outcome of interest with equal fidelity in different subgroups of patients. For example, in health care utilization research, the use of predicted health care *costs* underestimates health care *needs* in Black patients [8]. In the absence of performance-affecting bias, a classifier predicting high health care costs will have:


$$Q\left(X_{1},\;\hat{Y}\right)=Q\left(X_{2},\;\hat{Y}\right)$$

In other words, the performance may be equivalent between different groups, but the equivalent accuracy is due to underlying assumptions in the dataset.

For example, it may be the case that the White patients predicted to have high health care costs are, on average, less sick than the Black patients. The use of such a predictive model will lead to underallocation of health care resources to Black patients compared to their needs. We call this performance-invariant bias.

Let us assume without loss of generality that $\hat{Y}\left(x\right)=1$, when a model predicts the outcome of interest. Let $X_{a,h}$ and $X_{b,h}$ be the subsets of $X_{a}$ and $X_{b}$ respectively, consisting of patients predicted to have the outcome (like high health care costs). More formally,


$$X_{a,h}=\left\{x\in X_{a}\wedge \hat{Y}\left(x\right)=1\right\}$$

and


$$X_{b,h}=\left\{x\in X_{b}\wedge \hat{Y}\left(x\right)=1\right\}$$

We define a performance-invariant bias when $X_{a,h}$ and $X_{b,h}$ are not drawn from the same distribution.


$$X_{a,h}\neq X_{b,h}$$

To minimize performance-affecting bias, we need a new model $\hat{Y{\;}^{\prime }}$that is trained on more data (either more samples or more features) from the underrepresented population and reduces the discrepancy in the performance metric:


$$\left|Q\left(X_{a},Y,\;\hat{Y^{\prime }}\right)-Q\left(X_{b},Y,\;\hat{Y^{\prime }}\right)\right|<\left|Q\;(X_{a},Y,\hat{Y})\;-\;Q\left(X_{b},\;Y,\hat{Y}\right)\right|$$

To minimize performance-invariant bias, we must choose a different binary label $\hat{Z}\left(x\right)$ and develop a corresponding predictive model $\hat{Z(x)}$ such that if X is partitioned again based on the same sensitive characteristic, individuals predicted to be at higher risk are more similar to each other than they were with the label Y. More formally, the new predictive model gives us $X_{a,h}$ and $X_{b,h}$ as follows:


$$X_{a,h}\prime =\left\{x\;\in X_{a}\wedge \hat{Z}\left(x\right)=1\right\}$$

and


$$X_{b,h}\prime =\left\{x\;\in X_{b}\wedge \hat{Z}\left(x\right)\;=\;1\right\}$$

We expect our choice of Z to satisfy the following statement:


$$dis\left(X_{a,h}\prime ,\;X_{b,h}\prime \right)<\;dis\left(X_{a,h},X_{b,h}\right)$$

so individuals belonging to different sensitive categories but having the same outcome will now be more similar to each other. As an example, in the health care costs scenario, Black and White patients identified to be in the high-risk category will be more similar to each other than they were before.

We developed and validated AEquity, a machine learning–based method that can be used for the characterization of biases followed by guided mitigation in health care datasets (Textbox 1). AEquity works by appending a simple compressive network to a dataset or the latent space of existing machine learning algorithms to generate a single value, AEq, characterizing an outcome with respect to a group and the priors established by the algorithm. AEq is calculated by approximating a learning curve based on the model architecture, any pretrained weights, and a small sample of the dataset (<10%; supplementary methods are provided in Multimedia Appendix 1). After freezing the embedding layers, an autoencoder is trained on the embeddings instead of a classification block, like traditional transfer learning. We calculated AEq for each group by training the autoencoder block and estimating the loss function at each sample size. Next, we plotted the loss versus the sample size, fit a curve, and calculated the second derivative (supplementary code provided in Multimedia Appendix 1). In general, a higher AEq value for a group with respect to an outcome and a given machine learning algorithm suggests that generalization is more difficult and more or higher quality samples are needed for learning [19,20]. Thus, AEq is a metric that is calculated at the intersection of an outcome like “pneumonia,” a group label like “Black,” and a machine learning algorithm like “ResNet-50.” AEq is reported as $log_{2}\left(sample\;size\;estimate\right)$. AEq values are only meaningful when constrained to comparing groups with the same outcome in the dataset, and interpretation of the metric is invariant to the architecture of the machine learning algorithm, the compressive network, or the dataset type or characteristics [19,21,22].

Textbox 1. **AEquity-guided data collection of grouped objects.**

**Require:** {*x*_0,_*_m_*_,_*_y_*_,_ …, *x*_*n*,_*_m_*_,_*_y_*_,_ …, *x_N_*_,_*_m_*_,__*y*_} ∈ *X*, where *m* ∈ [1,2] refers to a specific population in the two distinct populations that *x* belongs to, *y* refers to the label that *x* has been assigned to, *n* refers to the data collected so far, and *N* is the total amount of data available for data collection under some resource or cost constraint. Let *Y*_₁_, *Y*_₂_, *Y*_₃_, …, *Y*_*K*_ ∈ **Y** refer to the set of all *K* possible measurable outcomes. Initialize a machine learning algorithm *f* with parameters *θ* that encodes *X* → *h*, classification block *c* that maps *h* → *y*, and decoder block *g* that maps *h* → *X*.

**for** *k* = 0 to *k* = *K* **do**

 **for** *m* = 0 to *m* = *M* **do**

  **for** *i* = 0 to *i* = *n* **do**

   *L*²_*i*,*m*,*k*_ ← *E*[(*x_i_*_,_*_m_*_,_*_k_* − *g*(*f*(*x_i_*_,_*_m_*_,_*_k_*)))²]

  **end for**

  *AEq*_*m*,*k*_ ← *d*² *L*²*_m_*_,_*_k_* / *n*²*_m_*_,_*_k_*

  *AEq_k_* ← *d*² *L*²*_k_* / n²*_k_*

 **end for**

**end for**

*Y*_optimal_ ← arg min_*Yk*_ *AEq*_1,*k*_ − *AEq*_2,*k*_

**if** *AEq_k_* < *AEq*_*m*,*k*_ for all *m* **then**

 Collect balanced data until *n* = *N*.

**end if**

**if** *AEq_k_* > *AEq_m_*_,_*_k_* for any m **then**

 **if** *AEq*_1,_*_k_* > *AEq*_2,_*_k_* **then**

  Prioritize collection of data from group 1 until *n* = *N*.

 **end if**

**end if**

To mitigate a specific type of bias, AEq values can be used to evaluate the right composition of the dataset for training a machine learning algorithm or for label selection without changing the dataset composition. To evaluate bias mitigation, we used an extensive variety of fairness metrics (area under the curve [AUC], true positive rate, false negative rate, true negative rate, positive predictive value, false positive rate, false discovery rate, and precision) and showed that AEquity can help improve these metrics over balanced empiric risk minimization.

**Figure 1.**
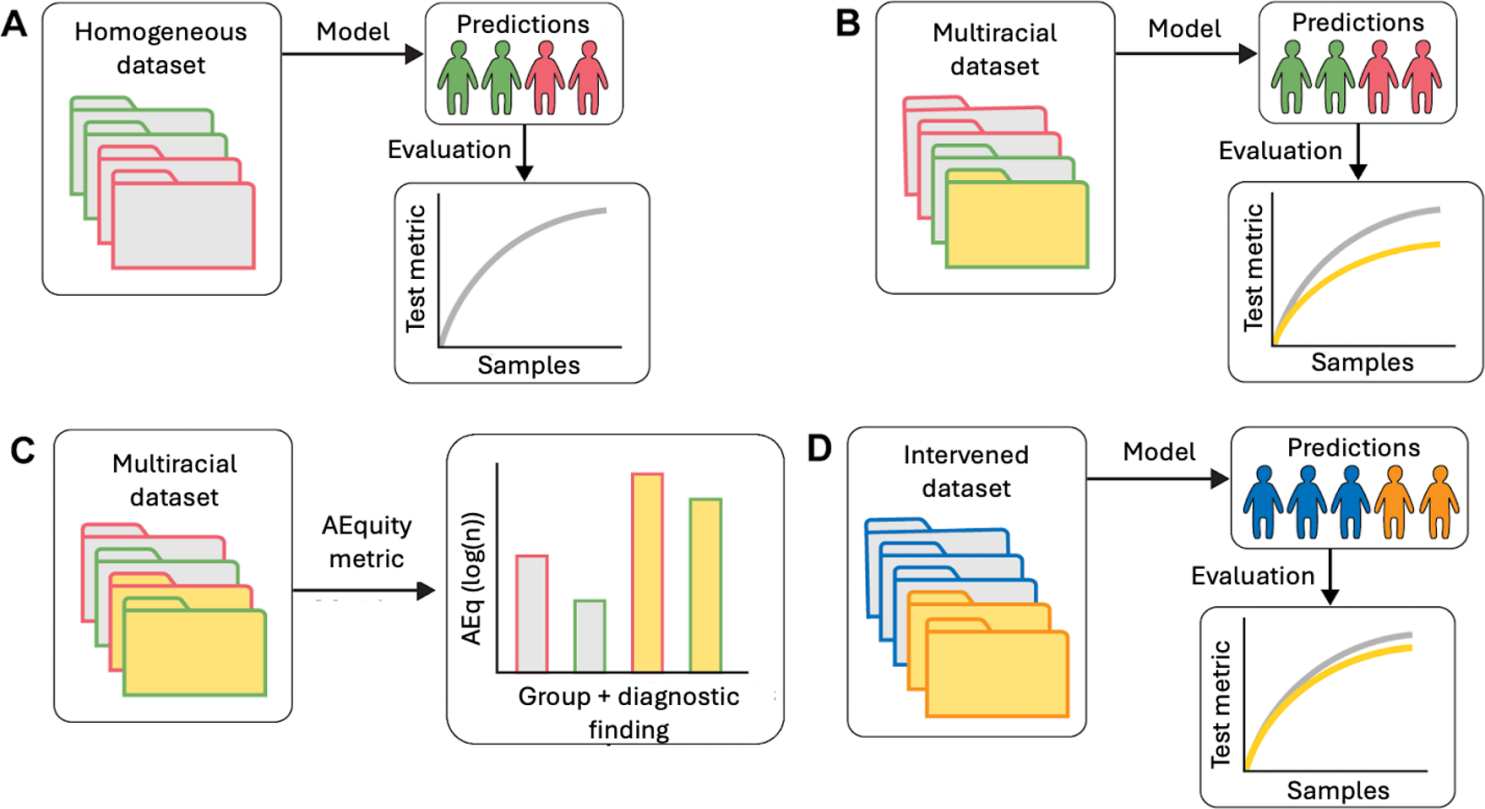
Schema of AEquity workflow to detect and mitigate diagnostic biases in the chest x-ray dataset. Gray and yellow represent White and Black subjects, respectively. Green and pink represent labels (diagnostic findings) in a dataset. (**A)** A model developed on a dataset consisting of White individuals and evaluated according to a chosen test metric. (B) A model developed on a dataset consisting of both White and Black individuals and evaluated according to the same test metric, showing a discrepancy in model performance for the two groups of subjects. (C) AEquity values (log(N)) by group and label. (D) Data-centric intervention based on the AEquity method reduced bias against Black patients.

AEquity-based data curation is achieved through a multistep pipeline (Figure 1, Textbox 1). First, a group-balanced subset of the patient data (with outcome labels) is collected. Next, AEq values by group for each outcome are calculated. The difference in the AEq values between groups for each outcome can help us determine if performance-invariant bias exists in the dataset. If AEq measures are significantly different between groups for the same label, then the label may not represent the same distribution for different groups. The label with least distributional difference (smallest difference in AEq values between groups) should be selected to mitigate the bias. Next, AEq is calculated with respect to each outcome when groups are combined into a joint dataset. The relationship between individual group and joint AEq values allows us to determine if sampling or complexity bias exists, and the differences in AEq values for different patient groups for the same label are an indicator of label bias. When predictive bias is driven primarily by sampling bias in the dataset, combining groups drives the AEq value at or below the oversampled data (supplementary methods are provided in Multimedia Appendix 1). In sampling bias, balanced sampling from each group is sufficient to mitigate the bias because the data are equitably represented within the algorithm. When complexity bias was the only type of dataset bias, combining the groups resulted in either an increase in the value of AEq or an AEq closer to a value that had been higher prior to the combination. In complexity bias, collecting data exclusively from the protected population, due to its relative heterogeneity compared to the overrepresented group, is necessary to mitigate bias. If the algorithm exhibits residual unfairness, then the AEq will be different for two groups for the same outcome. Thus, AEquity can help with quantifying biases within outcome metrics and subsequently selecting appropriate outcomes to minimize AEq values between groups. AEq values are all model- and task-specific and therefore no absolute values can be given. Instead, AEq values must be compared between groups and bootstrapping can help identify statistical significance. For all machine learning tasks, the training, validation, and test sets had no overlapping patients. The training and validation sets were seeded and bootstrapped 50 times. AEquity was applied to a small subset of the training data for all 3 tasks (<10%). The mean and standard errors were calculated across all 50 bootstraps and 95% CIs are reported. *P* values were calculated via *t* tests for 2-group quantitative analyses and ANOVA for multigroup quantitative analyses. All reported *P* values were adjusted with Bonferroni correction.

When training machine learning algorithms, all algorithms are trained on an independent training set and validated on a validation set and performances are reported on an independent test set (10%). The test set consists of held-out (nonoverlapping) samples prior to any dataset modification or intervention. Training and validation sets are bootstrapped 50 times, and performance is reported on the held-out test set. Means and 95% CIs are reported.

### Ethical Considerations

No informed consent was required because all data used in this publication were retrospectively collected, publicly available, and deidentified. The study was approved by the institutional review board at the Icahn School of Medicine at Mount Sinai.

## Results

### Detection and Mitigation of Racial Bias From Systematic Label Distortion in a Health Care Claims Dataset (Health Care Costs Scenario)

#### Background

In this example, we highlight the role of AEquity when labels for a particular group are systematically distorted for a given patient population. Obermeyer et al [8] showed that the use of healthcare *costs* as an outcome produced outcomes biased against Black patients, even though the classifiers predicted the costs equally well for both Black and White patients. This label bias could be mitigated by choosing an outcome that better measures health care needs. We examined whether AEquity can detect the biased outcome and help select a more equitable one. In this section, we clearly demonstrate that AEquity can be used at small sample sizes to effectively determine the outcome that minimizes biases from systematic label distortions.

**Figure 2.**
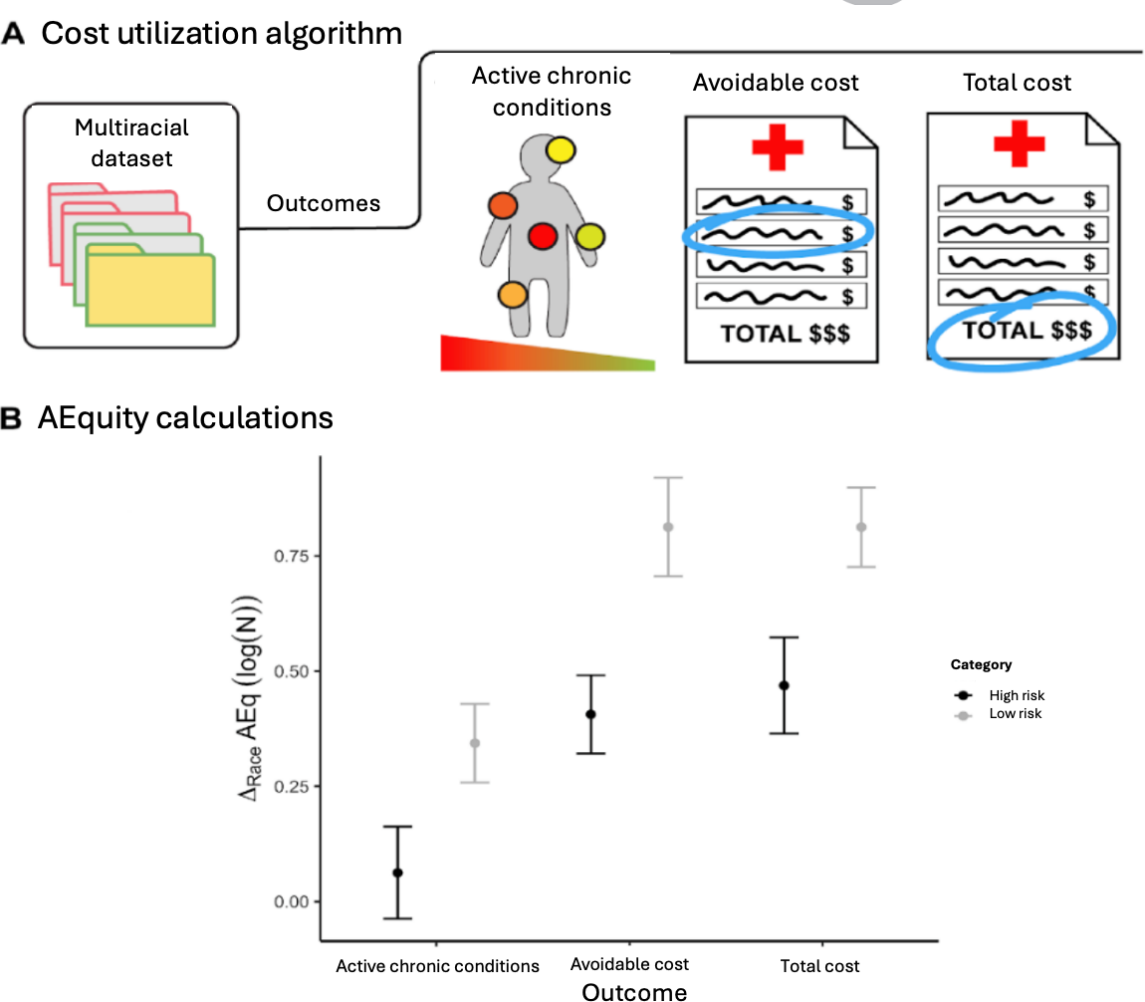
Detecting bias in the prediction of health care needs with the AEquity metric. (A) A dataset with claims data was used to calculate a score based on the number of active chronic conditions and two cost-based metrics. (B) The difference in AEquity values between Black and White patients for comorbidity- and cost-based outcomes, stratified by risk level.

First, a race-balanced subset of the patient data was collected (n=1024, 3% of the total available data); these data were labeled according to each of the available outcomes (active chronic conditions, avoidable costs, and total costs). We calculated AEq by race (Black, White), for each risk category (high risk, low risk), for each of the 3 outcomes (total costs, avoidable costs, active chronic conditions; Figure 2). To determine if Black and White patients in each risk category were similar, we then calculated the difference in AEq between races, for each risk category, for each outcome.

#### AEquity Can Guide Choice of Labels to Minimize Performance-Invariant Bias

First, the difference in AEq was statistically significant across outcome measures for the population designated as high-risk (ANOVA, *P*<.001). When predicted cost-based metrics (total costs, avoidable costs) were used to designate patients at highest risk, there was a significant difference in AEq between Black and White patients (Figure 2). For example, with total costs, the difference was 0.47 (95% CI 0.36-0.52). Thus, Black patients predicted to be at high risk were significantly different from their White counterparts, which led to inequitable resource allocation. However, the difference between Black and White patients categorized to be at high risk disappeared when using predicted active chronic conditions (instead of predicted costs) as the outcome measure; the difference in AEq was not statistically significantly different from 0 (95% CI −0.04 to 0.16), indicating that Black and White patients in that risk category were similar. Thus, AEq values could a priori guide the choice of a better outcome measure to identify patients at high risk and thus mitigate label bias.

We conducted additional analyses in patients at lower risk and found that the differences in AEq across races were all statistically significant (Figure 2B). In the low-risk group, Black patients displayed more heterogeneous underlying characteristics than White patients. We also observed that the differences in the AEq metric were smaller when using active chronic conditions compared to using avoidable costs or total costs, indicating mitigation of label bias for this lower-risk group despite the increased heterogeneity (Table 1). Nevertheless, the presence of differences in AEquity score in the low-risk group may indicate that there may be some residual complexity or sampling bias even after changing the outcome metric.

**Table 1.** Comparing our models used for AEquity experiments to state-of-the-art methods on chest X-ray classification tasks.

Method	Test area under the receiver operating characteristic curve
RadImageNet [23]	0.71
Wang et al [24]	0.74
Ho et al [25]	0.74
Yao et al [26]	0.78
Ours (RadImageNet)	0.74
Ours (Vit_B_16)	0.83

### Detection at Small Sample Sizes and Mitigation of Performance-Affecting Bias in Chest X-Rays (Chest Radiographs Scenario)

#### Background

In this section, we demonstrate that AEquity can be used at small sample sizes to guide data collection at larger sample sizes to mitigate performance bias between predetermined patient populations. We followed a multistep pipeline for AEquity-based data curation. First, we collected a balanced subset of the patient data (n=1024, 3% of the total available data, 512 Black patients, 512 white patients) and labeled it according to each of the available labels. For each diagnostic finding, we calculated AEq by race (Black, White) and also for the joint dataset containing samples from both Black and White patients. We then used these AEq values to guide data curation, choosing either (1) to group-balance with an equal number of additional samples from both Black and White patients or (2) to prioritize data collection of a single group to a total of 30,000 samples, a sample size sufficient to produce performance comparable to state-of-the-art classification algorithms.

We took a data-centric approach with simple training of a ResNet-50 pretrained model and achieved similar performance to state-of-the-art approaches that use a ResNet-50 backbone (Table 1).

We then demonstrated that AEquity can help analyze causes of sampling and complexity bias at both small and large dataset sizes, but it becomes more precise at larger sample sizes (Figure S1 in Multimedia Appendix 1). We split the subset 60%‐20%‐20% into training samples, validation samples, and test samples, ensuring no patients overlapped, and fine-tuned two different pretrained machine learning algorithms (ResNet-50 and VIT-B-16) with PyTorch Lightning [23,27,28]. First, we compared these algorithms to state-of-the-art chest X-ray classification algorithms (Table 1).

**Figure 3.**
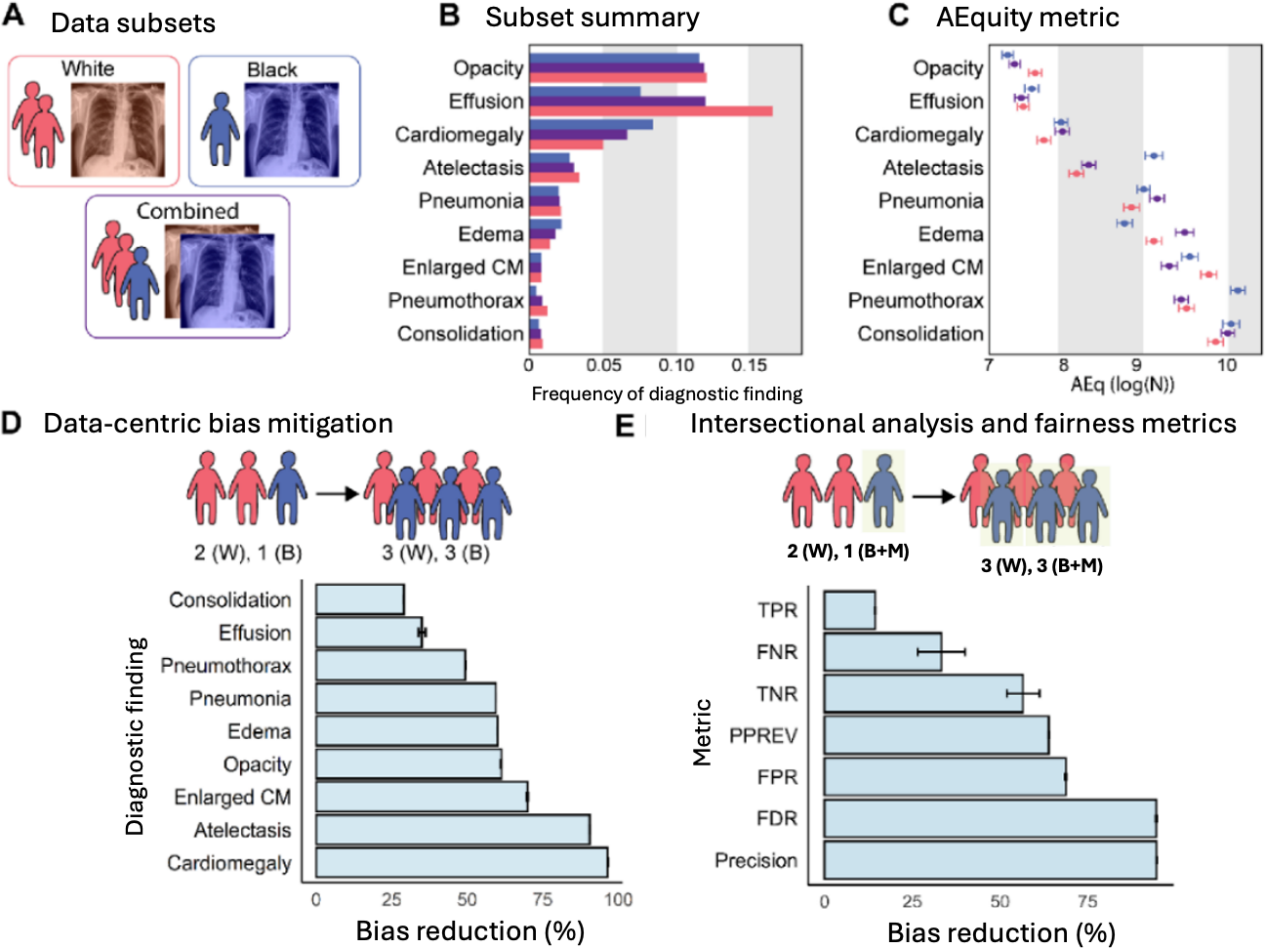
Application of AEquity to detect and mitigate diagnostic biases in the chest x-ray dataset. (**A**) Red represents White individuals and blue represents Black individuals. (B) Frequency of diagnostic findings by race. Purple represents the average of White and Black individuals. (C) AEquity values (log(N)) by diagnosis and race. (D) Effect of data-centric mitigation intervention on bias against Black patients. (E) Decrease in bias as measured by different fairness metrics in Black patients on Medicaid. B: Black; CM: cardiomediastinum; FDR: false discovery rate; FNR: false negative rate; FPR: false positive rate; M: Medicaid; PPREV: positive predictive value; TNR: true negative rate; TPR: true positive rate; W: White.

Next, we showed how AEquity can improve performance over naive data collection (Figure 3, Table 2) and benchmarked against balanced empirical risk minimization, which has previously been shown to outperform other variants of subgroup characterization such as Just-Think-Twice and GroupDRO (Distributionally Robust Optimization) on chest X-ray datasets (Table 3, Figure S2 in Multimedia Appendix 1).

**Table 2.** Measurement of bias and intervention effect by diagnosis in the radiograph dataset with RadImageNet (ResNet-50 with pretraining on medical images).

Diagnosis	Preintervention, mean (95% CI)			AEquity-guided, mean (95% CI)			Bias reduction, mean (95% CI)
	White patients	Black patients	Bias	White patients	Black patients	Bias	
Pneumothorax	0.73 (0.72-0.74)	0.68 (0.67-0.69)	7.5	0.73 (0.72-0.74)	0.70 (0.68-0.72)	3.8	49.4 (49.3-49.5)
Atelectasis	0.69 (0.68-0.69)	0.64 (0.64-0.65)	6.4	0.70 (0.70-0.70)	0.69 (0.69-0.69)	0.6	90.6 (90.5-90.7)
Edema	0.75 (0.75-0.75)	0.72 (0.71-0.72)	5.0	0.78 (0.78-0.78)	0.79 (0.79-0.80)	2.0	60.0 (59.9-60.1)
Consolidation	0.67 (0.67-0.67)	0.65 (0.64-0.66)	3.8	0.63 (0.63-0.63)	0.61 (0.60-0.63)	2.8	29.0 (28.8-29.2)
Pneumonia	0.67 (0.66-0.67)	0.64 (0.64-0.65)	3.8	0.65 (0.65-0.65)	0.64 (0.64-0.65)	1.5	59.5 (59.4-59.6)
Enlarged cardiomediastinum	0.62 (0.61-0.63)	0.60 (0.59-0.60)	3.6	0.61 (0.61-0.62)	0.61 (0.60-0.61)	1.1	69.9 (69.6-70.1)
Cardiomegaly	0.76 (0.76-0.76)	0.75 (0.75-0.75)	1.9	0.76 (0.76-0.76)	0.76 (0.76-0.76)	0.1	96.5 (96.4-96.6)
Pleural effusion	0.64 (0.64-0.64)	0.64 (0.64-0.64)	0.3	0.65 (0.65-0.65)	0.65 (0.65-0.65)	0.2	35.0 (33.7-36.2)
Opacity	0.69 (0.69-0.69)	0.71 (0.70-0.71)	–2.0	0.69 (0.69-0.69)	0.70 (0.69-0.70)	–0.8	61.2 (61.0-61.4)

**Table 3.** Measurement of bias and intervention effect by diagnosis in the radiograph dataset with vision transformers.

Method	Naive data collection, AUROC^a^ (95% CI)		Balanced empirical risk minimization, AUROC (95%CI)		AEquity-guided, AUROC (95% CI)	
Label	White	Black	White	Black	White	Black
Opacity	0.8126 (0.8102-0.8149)	0.8154 (0.8131-0.8178)	0.8139 (0.8119-0.8159)	0.8169 (0.8149-0.8188)	0.8126 (0.8102-0.8149)	0.8154 (0.8131-0.8178)
Effusion	0.925 (0.9242-0.9257)	0.9195 (0.917-0.9221)	0.9249 (0.9239-0.9259)	0.9192 (0.9164-0.922)	0.925 (0.9242-0.9257)	0.9195 (0.917-0.9221)
Cardiomegaly	0.8613 (0.8603-0.8624)	0.8611 (0.8596-0.8625)	0.864 (0.8625-0.8654)	0.8647 (0.8632-0.8662)	0.8633 (0.8622-0.8644)	0.8632 (0.8619-0.8645)
Atelectasis	0.8512 (0.8497-0.8527)	0.8463 (0.8431-0.8495)	0.8508 (0.8496-0.852)	0.8457 (0.8432-0.8481)	0.8426 (0.8413-0.8439)	0.8396 (0.8375-0.8418)
Pneumonia	0.7781 (0.7769-0.7792)	0.771 (0.7677-0.7743)	0.7772 (0.7754-0.7791)	0.7706 (0.7671-0.7741)	0.7782 (0.7769-0.7794)	0.7836 (0.7803-0.7869)
Edema	0.9276 (0.9263-0.9289)	0.9303 (0.9278-0.9327)	0.925 (0.9226-0.9274)	0.9286 (0.9254-0.9318)	0.9276 (0.9263-0.9289)	0.9303 (0.9278-0.9327)
Enlarged cardiomediastinum	0.6948 (0.687-0.7025)	0.6591 (0.6422-0.6761)	0.6974 (0.6898-0.7049)	0.6588 (0.6412-0.6763)	0.6948 (0.687-0.7025)	0.6591 (0.6422-0.6761)
Pneumothorax	0.818 (0.8114-0.8247)	0.7439 (0.7123-0.7755)	0.8139 (0.8063-0.8215)	0.7408 (0.7103-0.7713)	0.8075 (0.7995-0.8155)	0.7374 (0.708-0.7668)
Consolidation	0.7903 (0.7847-0.7958)	0.7372 (0.7145-0.76)	0.7897 (0.7847-0.7947)	0.7361 (0.7136-0.7587)	0.7948 (0.7896-0.7999)	0.7443 (0.7213-0.7674)

a AUROC: area under the receiver operating characteristic curve.

#### AEquity Can Help Diagnose and Mitigate Bias Based on Demographic Characteristics in a Chest X-Ray Dataset

Table 3 shows the AUROC before and after intervention with 95% CIs for Black and White patients. Performance bias is the difference in the AUROC between Black and White patients calculated in percentages. With a dataset intervention, we reduced the discrepancy between the two groups and calculated the percentage change in the discrepancy. Percentages have been rounded to 1 decimal place, and all other numbers have been rounded to 2 decimal places. The asterisk indicates diagnostic findings with bias against Black patients.

We validated against threshold-independent metrics such as AUC as well as fairness metrics (Table 4). We bootstrapped each experiment 50 times, in keeping with available computational resources. We defined bootstrapping as sampling with replacement, but avoided sampling patients and their chest X-rays from the test dataset.

**Table 4.** Measurement of bias and intervention effect by metric on an intersectional population.

Metric	Bias reduction (% [95% CI])
True positive rate	14.4 (14.3-14.5)
True negative rate	56.6 (51.9-61.3)
False positive rate	68.7 (68.4-69.0)
False negative rate	33.3 (26.6-40.04)
Precision	94.6 (94.5-94.7)
False discovery rate	94.5 (94.3-94.7)

We show the effect of the interventions on fairness metrics for a patient population that is both Black and on Medicaid compared to White patients (Figure S5 in Multimedia Appendix 1). With a dataset intervention, we have reduced the discrepancy between the two groups.

Performance bias for each diagnostic finding in the chest radiograph dataset decreased by between 29% and 96.5% following intervention. Consequently, we demonstrated that (1) AEquity remains valid for mitigation of performance bias by sex, (2) AEquity-guided data curation is effective for individuals at the intersection of two or more disadvantaged groups, (3) targeted data collection and curation is robust to choice of fairness metric, and (4) these results are robust when trained on transformer architectures with 86 million parameters. When evaluating sex, the joint AEq score for all 9 diagnostic findings was not significantly different from or lower than the AEq score for females, indicating that predictive biases are primarily driven by sampling bias (Figure S3 in Multimedia Appendix 1). Larrazabal et al [16] previously demonstrated that group balancing is the optimal approach to mitigate gender biases in this setting. Second, we demonstrate the effectiveness of AEquity on intersectional populations, who are at greater risk of being affected by systemic biases [29], and we show that this technique is robust to the choice of fairness metrics. When we examined Black patients on Medicaid, at the intersection of race and socioeconomic status, we found that AEquity-based interventions reduced the false negative rate by 33.3% (absolute reduction =1.88×10^−1^, 95% CI 1.4×10^−1^ to 2.5×10^−1^); precision bias by 94.6% (absolute reduction 7.50×10^−2^, 95% CI 7.48×10^−2^ to 7.51×10^−2^); bias in false discovery rate by 94.5% (absolute reduction=3.50×10^−2^, 95% CI 3.49×10^−2^ to 3.50×10^−2^) (Figure S5 in Multimedia Appendix 1). In this example, AEquity functions by recommending data collection for more Black patients, more patients on Medicaid, or more Black patients on Medicaid depending on the relative AEq values for each group as previously described. Finally, we demonstrated AEquity works on VIT-B-16, a vision transformer with 86 million parameters (Figure S2 in Multimedia Appendix 1). Notably, in 4 of the 9 diagnostic findings (opacity, effusion, edema, and enlarged cardiomediastinum), analysis of AEquity values resulted in the curation of a dataset reflecting the underlying empirical group frequencies. However, in the remaining 5 (cardiomegaly, atelectasis, pneumonia, pneumothorax, and consolidation), AEquity led to a dataset that prioritized one group, outperforming balanced empirical risk minimization for the mitigation of sampling and complexity bias.

We did not examine label bias with chest radiographs due to the unavailability of gold-standard labels besides those already present in the publicly available dataset. However, Zhang et al [9] have previously shown that, at least for the “No Finding” label in the chest radiograph dataset, the prevalence of incorrect labeling did not vary significantly by race and sex, but that it was more prevalent in the subgroup of patients over 80 years of age.

### Detection of Previously Unknown Bias in the National Health and Nutrition Examination Survey for Predicting All-Cause Mortality (NHANES Scenario)

#### Background

In this section, we used AEquity to detect a novel sampling, complexity, and label bias in a widely used dataset (National Health and Nutrition Examination Survey [NHANES]) for a common task (mortality prediction) with a popular nondifferentiable model (Light Gradient-Boosting Machine). The NHANES data were collected between 1999 and 2014 and include demographic, laboratory, examination, and questionnaire features. The NHANES dataset has been used for a wide range of applications including the measurement of the population prevalence of metabolic syndrome, demographic trends in major depressive disorder, and associations between factors such as inflammation and autoimmune diseases [30,31]. Machine learning has been used on the NHANES dataset to predict coronary heart disease, hypertension, and all-cause mortality [32-34].

**Figure 4.**
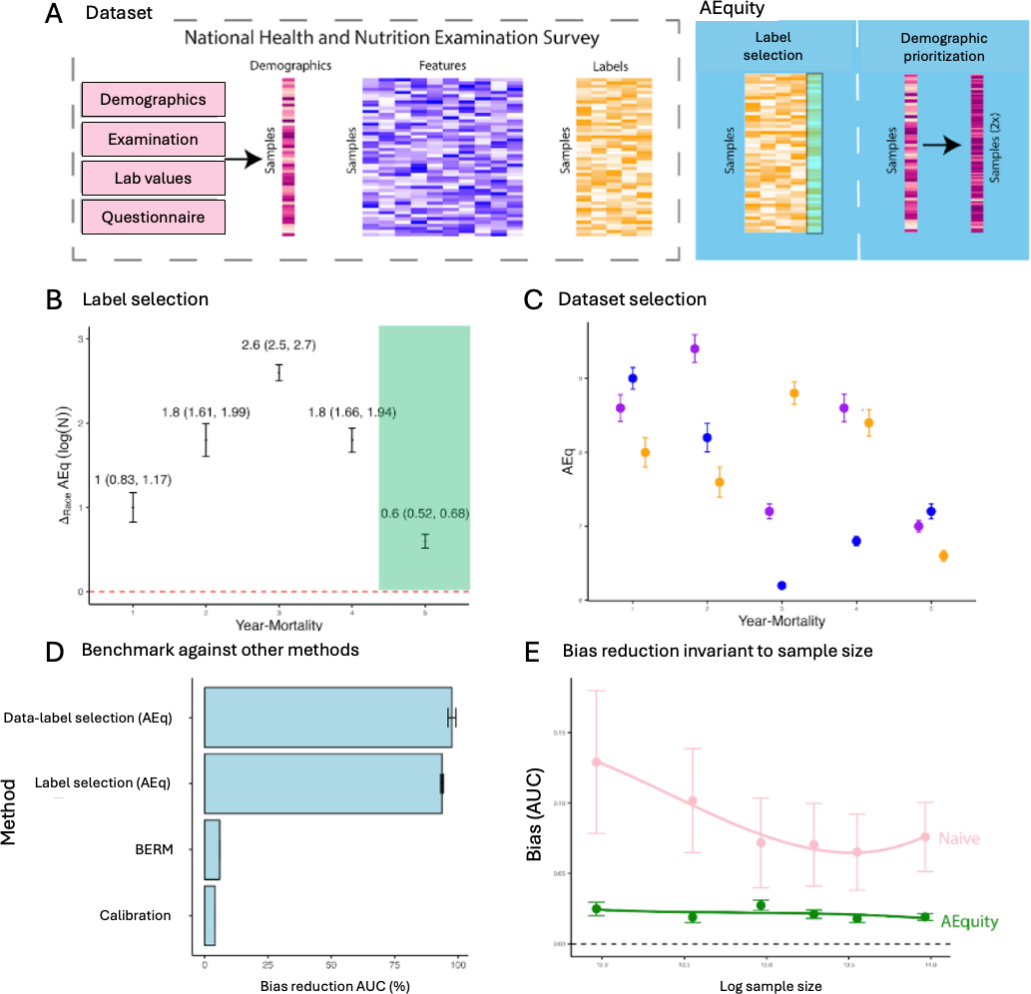
Application of AEquity to detect, characterize, and mitigate a previously undetected racial bias in mortality prediction on a commonly used dataset. (**A)** Schematic representations of AEquity applied to label selection and demographic prioritization of bias in the National Health and Nutrition Examination Survey. (**B)** AEquity reveals that the least biased label for mortality prediction occurs at 5 years because the groups have similar levels of complexity. (**C)** AEquity determined that 5-year mortality has complexity bias because the joint AEq exceeds the AEq value of both Black and White patients. (D) AEquity outperformed other modalities of bias reduction, namely BERM, and calibration. (E) AEquity demonstrated that bias reduction is invariant to sample size. AUC: area under the curve; BERM: balanced empirical risk minimization.

The prediction of all-cause mortality is an important task because all-cause mortality is a commonly chosen outcome in epidemiological studies [35]. All-cause mortality can be used as a metric for health disparities, to guide public health interventions, and to affect health resource allocation [36-38]. Therefore, detecting, characterizing, and mitigating sampling, complexity, and label biases in the prediction of all-cause mortality can potentially affect large-scale epidemiological interventions. To evaluate these biases in the prediction of all-mortality in the NHANES dataset, we preprocessed the data (N=47,261) and selected variables as described in past work by Qiu et al [34]. To detect potential sampling, complexity, and label biases, we calculated AEq values at small sample sizes (N=400) for Black and White individuals and all-cause mortality for different follow-up periods in the NHANES dataset (Figure 4A). Next, we qualitatively and quantitatively analyzed the AEquity values to identify the types of biases in the dataset. Finally, we used AEquity to guide a data-centric intervention and compared this intervention with simple algorithmic calibration and balanced empirical risk minimization. All experiments were bootstrapped 50 times with the Pytorch library.

#### AEquity Enables Detection and Mitigation of Performance-Affecting and Performance-Invariant Bias

First, we evaluated for label bias by calculating the difference in AEquity score between Black and White patients for all-cause mortality at different follow-up periods (Figure 4B). AEquity scores for Black and White patients are least significantly different at 5-year mortality (AEq_Difference, 5-year_=0.60, 95% CI 0.52-0.68; AEq_Difference, 1-year_=1.00, 95% CI 0.83-1.17; *P*<.05). Next, we evaluated for complexity and sampling bias based on 5-year-mortality and race (Figure 4C). The AEq value for the joint distribution was significantly larger than the AEq values for either Black or White patients, signaling complexity bias (AEq_Joint, 5-year_=7.00, 95% CI 6.93-7.07; AEq_White, 5-year_=7.00, 95% CI 6.93-7.07; AEq_Black, 5-year_=6.6, 95% CI 6.53-6.66; *P*<.05).

Based on the results from AEquity, we curated a dataset with prioritized data from Black patients, trained a gradient boosted tree to predict 5-year mortality, and compared its fairness metrics to that of a gradient boosted tree trained to predict 1-year mortality with a dataset sampled based on population prevalence. First, AEquity significantly reduced performance bias as measured by the difference in AUC between Black and White patients and outperformed both calibration and balanced empirical risk minimization (Figure 4D). Second, label selection and dataset prioritization had synergistic bias mitigation effects (Figure 4D). Third, AEquity’s performance bias mitigation relative to training with random sampling from the original dataset is invariant to sample size (Figure 4E).

## Discussion

### Principal Findings

In summary, we demonstrated that AEquity is a task-, algorithm-, and architecture-agnostic machine learning–based metric that may be valuable for disentangling and quantifying various types of biases at the dataset level.

Algorithmic bias is one of the major challenges to the adoption of artificial intelligence in health care and is a focus for regulation. For example, the Good Machine Learning Practice guidance from the Food and Drug Administration and European Medicines Agency emphasizes the importance of ensuring that datasets are representative of the intended patient population [39]. However, this is not sufficient because significant bias can result from the choice of outcome or differences in group complexity even when datasets are diverse. AEquity, the machine learning–based technique described in this paper, can help characterize and mitigate bias early in the algorithm development life cycle.

The responsibility for ensuring algorithmic equity is being placed on algorithm developers and institutions. The US Department of Health and Human Services has proposed a rule under Section 1557 of the Affordable Care Act to ensure that “a covered entity must not discriminate against any individual on the basis of race, color, national origin, sex, age, or disability through the use of clinical algorithms in decision-making.” More recently, an executive order has placed at least some of the liability for algorithmic bias on developers and deployers of these algorithms [40]. Therefore, the need to develop tools to identify and remediate potential bias in health care datasets is urgent from both ethical and regulatory perspectives. The recently developed European Union artificial intelligence act (EU AI Act) requires rigorous testing and validation to detect model bias, including specific provisions for data governance [41].

The algorithm development pipeline involves task identification, data collection, data curation, training, validation, and deployment, each of which plays an important role in whether it adds value to the health care system or propagates the disparities that it seeks to address [42]. The ability of this approach to quantify and address bias at small sample sizes makes it an attractive addition to the tool kit, akin to sample size determination but for bias [43]. Nevertheless, data-centric curation is only one of many approaches in which bias can be mitigated.

Currently, the most prevalent solution for bias mitigation with data-centric approaches is the comparison of predictive power across protected groups and balanced empirical risk minimization. We have demonstrated the application of AEquity to datasets where biases have been confirmed and described in high-quality peer-reviewed studies, but application of the current state-of-the-art method falls short. Algorithms predicting all 3 labels in the health care costs scenario of Obermeyer et al [8] have similar predictive power, but 2 of the 3 are biased, demonstrating that equal predictive power is not a reliable indicator of the absence of bias. AEquity can identify the biased labels by examining a small subset of the data. Second, we demonstrated in the chest radiograph scenario that the optimal mitigation of bias sometimes requires the prioritization of data from the minority population and at other times, it requires a larger, more balanced dataset. AEquity can help distinguish between these two scenarios early in the data collection process, because it only requires a small amount of data for analysis. Third, we used AEquity to describe and mitigate a previously undetected and unreported bias in all-cause mortality prediction based on data from the NHANES. Applying AEquity to the NHANES dataset can detect the bias at small sample sizes and help optimize label selection and dataset curation to mitigate bias across a range of sample sizes.

Data-guided strategies face challenges due to the lack of representation in many datasets [44,45]. Moreover, once a gap has been identified, targeted data collection of protected populations raises ethical concerns given the long history of mistrust [46]. Nevertheless, acknowledging the existence of these biases through quantitative measures can build trust, a crucial step toward mitigating some of these biases at a grassroots level [47,48]. Investigating the reason why AEq scores are different between different populations may provide key insights into underlying mechanisms of racial inequities. AEquity provides insight into key discrepancies that can occur at intersectional identities, not just between single groups. AEq can help guide sampling strategies early in the data collection process because it requires relatively few samples. In past work, we highlighted that the sample size at which the training loss curve of an autoencoder converges corresponds to the sample size at which a training loss curve for a neural network classifier trained on the same data converges. We showed that this holds in a variety of computer vision datasets and on latent spaces belonging to a variety of structures, which included varying the number of classes, number of informative features, number of total features, number of class-specific subgroups, and the relationships between different variables within the dataset. In this work, we build on that approach to the problem of bias mitigation in health care. Here, we do not use it to predict sample size for training a neural network. Rather, we show that it can be used as a simple marker of the learnability of data subsets and to further guide dataset intervention, which can reduce the bias of classifiers trained on the data. The demonstration of AEquity with multiple state-of-the-art algorithms, multiple tasks, and multiple metrics provides strong validation for its use in practice.

### Limitations and Future Work

Although our results are promising, there are some limitations to AEquity and directions for future inquiry. In the following sections, we discuss four key limitations: the potential costliness of data collection, the need to expand equity beyond classification tasks, the need to clearly identify gold-standard labels, and the structural inability for AEquity to distinctly pinpoint underlying causes for dataset bias.

We acknowledge that in health care, collecting data can be difficult and costly. Additional approaches are being developed to help improve data availability for subgroups via generative models [49]. However, this does not necessarily replace high-quality data collection because large models are at the risk of collapsing when trained on recursively generated data [49,50]. Moreover, the cost of guiding data collection to minimize bias may be offset by cost reduction in easing approval through regulatory organizations [39].

Second, AEquity in its current form focuses primarily on classification tasks. A growing number of algorithms, however, are (1) using various forms of regularization and attention to produce more informative latent spaces [51], (2) being used for generation rather than simply classification or regression [51,52], and (3) multimodal. However, we have demonstrated that AEquity is invariant to the model architectures that underpin many of the multimodal and generative models such as convolutions and transformers. Nevertheless, further work should be done to demonstrate invariance to task selection.

Third, if AEquity identifies systematic label distortion, then new gold-standard labels are required. A range of different post hoc fairness metrics can be violated if the labels are systematically biased [53]. As a result, new labels may be required. However, obtaining gold-standard labels requires domain expertise and can be costly and time-intensive. Further work should focus on how to mitigate bias when only a few gold-standard labels are readily available.

Finally, if the dataset domain does not contain underlying information about the origins of bias, AEquity is limited in the types of conclusions that it can draw. For example, AEquity can help characterize the bias as primarily complexity-related, which may lead an investigator to hypothesize that it may be due to more advanced disease due to lack of access to care. Yet, without explicit information on lack of access to care, AEquity cannot confirm that lack of access to care is indeed the cause. However, if the investigator adds lack of access to care as a latent variable, AEquity may be able to identify types of biases related to lack of health care access. Future work should explore how adding latent variables directly affects AEquity’s explainability.

One of the key questions that remains is generalizability. In this paper, we demonstrated that AEquity empirically works across a range of different dataset sizes, data modalities, and model architectures. For example, we demonstrated that AEquity works on a small number of samples (N=256) with models that have relatively few parameters and are nondifferentiable (Light Gradient-Boosting Machine), as well as larger sample sizes (N=30,000) with models that have up to 86 million parameters including convolutional and transformer architectures. We have demonstrated that AEquity functions across different data modalities (image and tabular), as well as across different performance metrics (AUROC, false discovery rate, false negative rate) and for multiple intersectional subgroups (Black patients and patients of low socioeconomic status). Moreover, we have additionally included the code for other individuals to continue testing AEquity across a range of different datasets and model parameters. Nevertheless, in models that exhibit emergent properties such as large language models with billions of parameters, AEquity needs to be continually evaluated to identify if emergent properties can affect the intrinsic representation of bias within a model.

In summary, we present AEquity, a task-, algorithm-, and architecture-agnostic machine learning–based metric that may be valuable for disentangling and quantifying various types of biases at the dataset level. Most importantly, we have identified that AEquity is functional in the most common deep learning methods (transformers and convolutions) and the most common nonparametric method (gradient boosted machines). We have demonstrated that it can reduce biases across different underserved populations (Black patients) and at the intersection of underserved populations (Black patients on Medicaid). A key implication of this work could be broad-spectrum application to datasets on which algorithms are trained prior to deployment as a key regulatory step for the Food and Drug Administration and the European Medical Services [8,54-58].

## Supplementary material

10.2196/71757 Multimedia Appendix 1 Supplementary Figures S1-S5 and supplementary methods.

## Abbreviations

AUC: area under the curve
AUROC: area under the receiver operating characteristic curve
NHANES: National Health and Nutrition Examination Survey
