# Supplementary material for: Detecting, Characterizing, and Mitigating Implicit and Explicit Racial Biases in Health Care Datasets With Subgroup Learnability: Algorithm Development and Validation Study
Source: J Med Internet Res. 2025 Sep 4;27:e71757. doi: 10.2196/71757 (PMC12410029; doi:10.2196/71757)
Supplement: Multimedia Appendix 1 [file jmir-v27-e71757-s001.docx]

# Appendix

### **Supplementary Figure S1. AEquity remains functional at smaller sample sizes.**


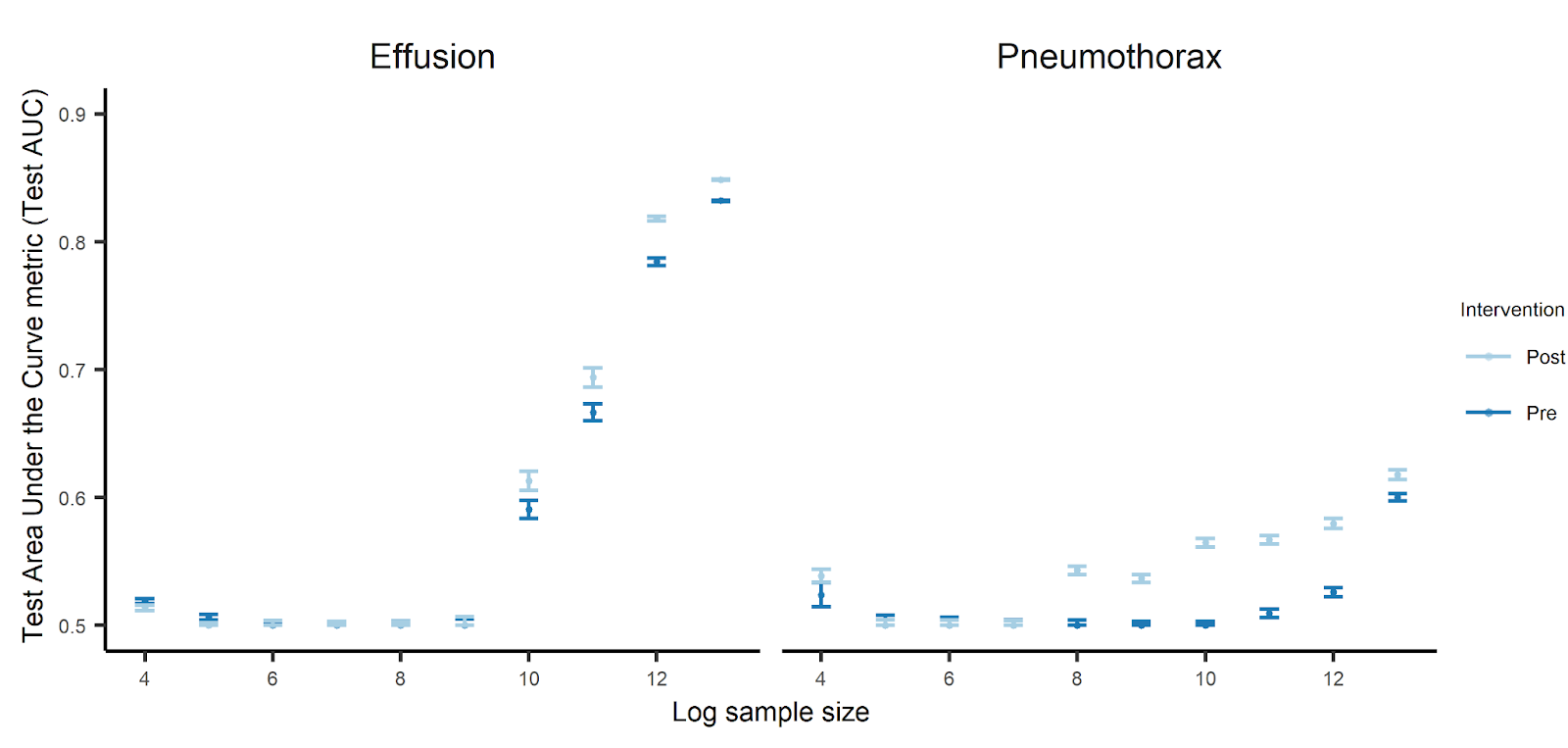

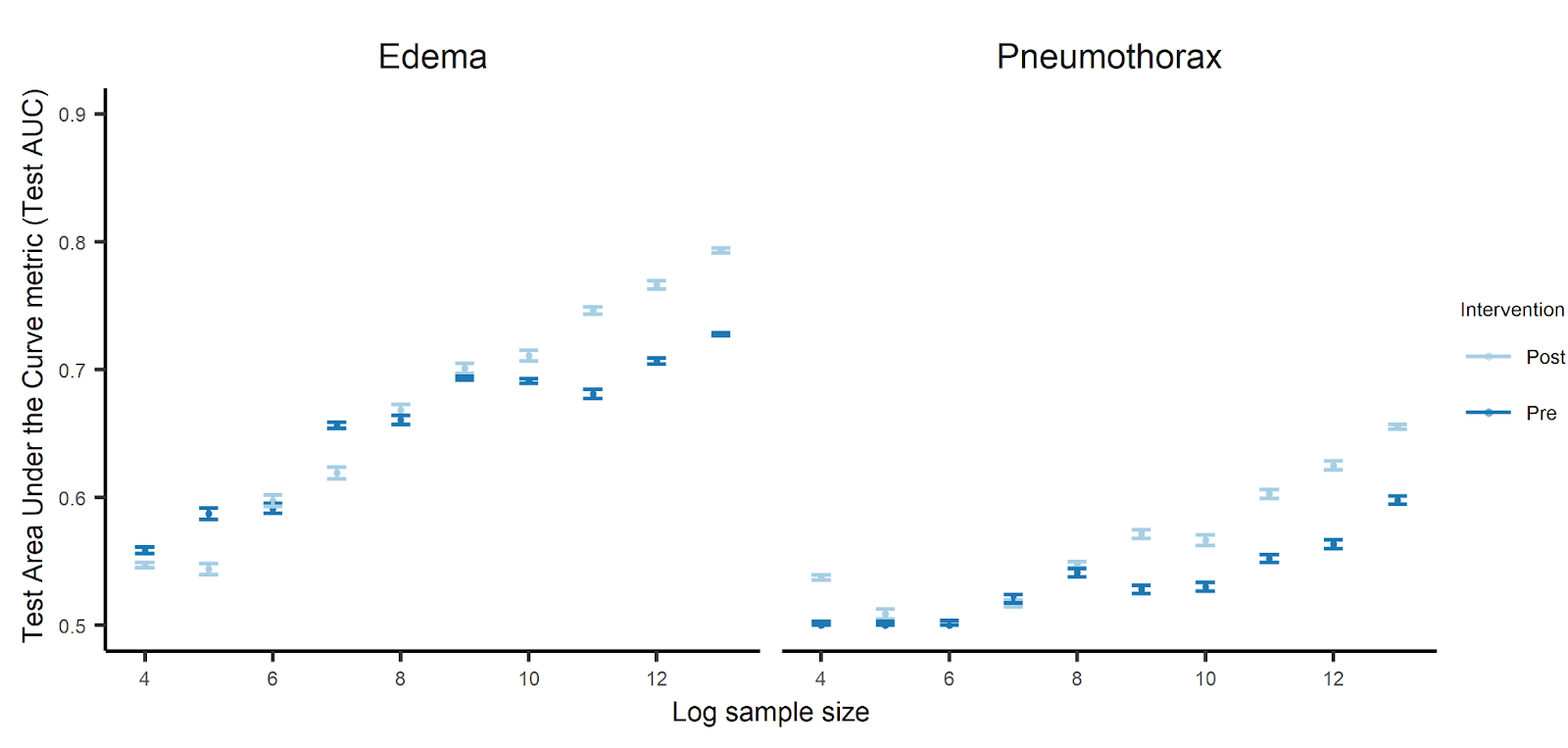


**Supplementary Figure S1.** AEquity becomes increasingly precise as the number of samples increases and generalizes across different sample sizes when using small convolutional neural networks.

### **Supplementary Figure S2. Benchmarking AEquity against Balanced ERM in Vision Transformers as measured by Area-Under-the-Curve**


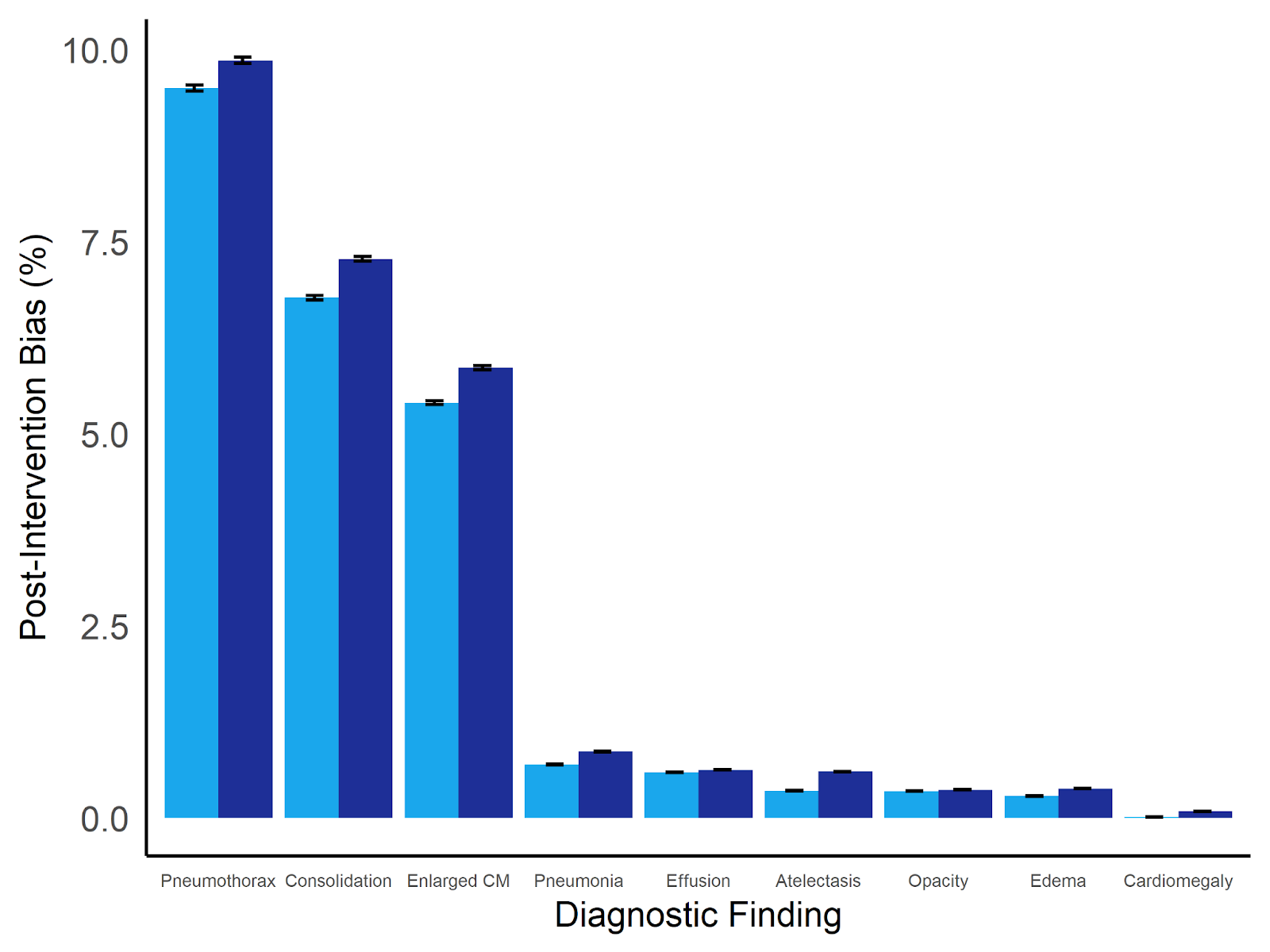


**Supplementary Figure S2.** Measurement of bias by diagnosis in the radiograph dataset with VisionTransformers (86 M parameter VIT_B_16 with pre-training on IMAGENET) via area-under-the-curve. Light-blue is AEquity, Dark Blue is Balanced Empirical Risk Minimization.

### **Supplementary Figure S3**. Replication on sex demographic


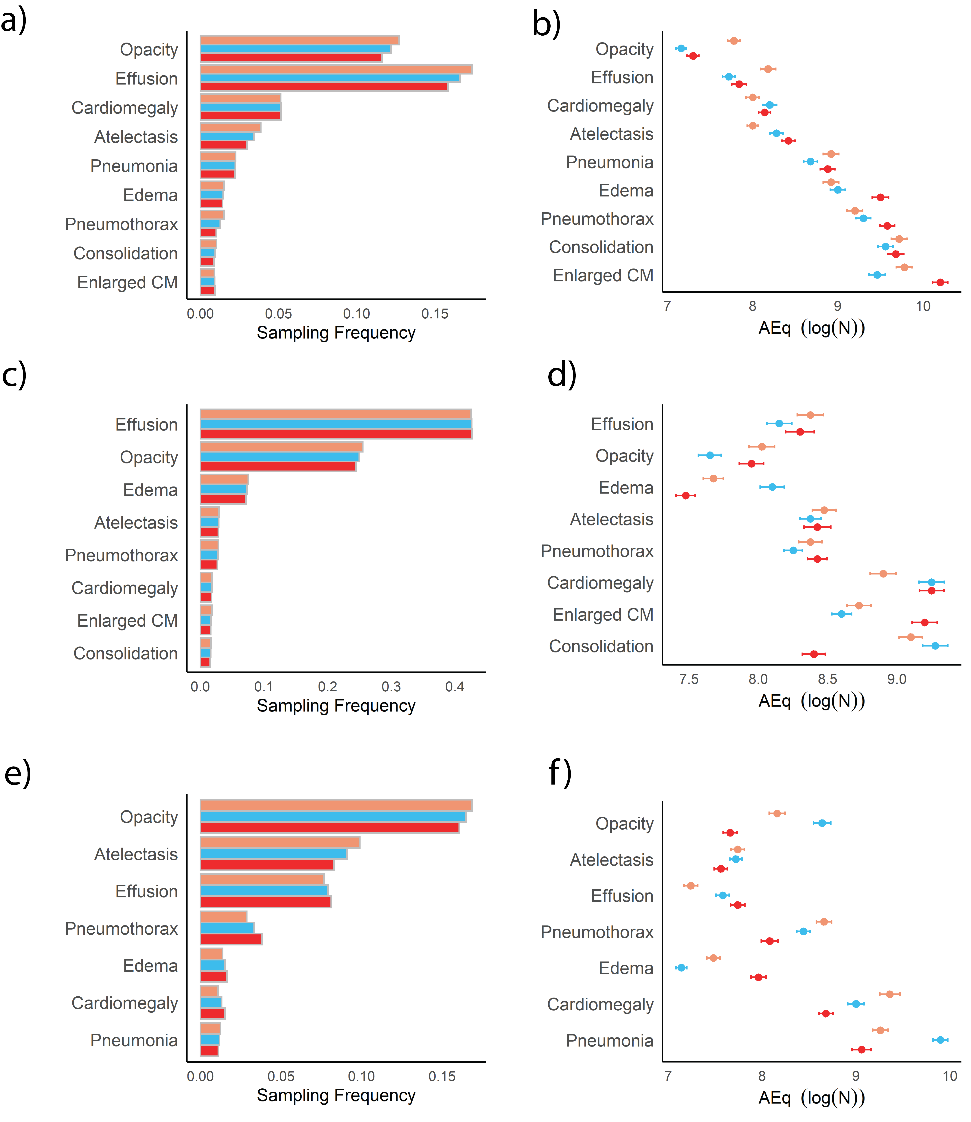


**Supplementary Figure S3**. **Replication on sex demographic**. Red is female, orange is male and blue is the combined dataset. **a)** Histogram by class label of MIMIC-CXR and **b)** AEquity values for MIMIC-CXR. (R = -0.80, P = 4.94 x 10^-7^) **c)** Histogram by class label for CheXPert and **d)** AEquity values by class label for CheXPert. (R = -0.41, P = 8 x 10^-3^). **e)** Histogram by class label for ChestX-ray14, and **f)** Corresponding AEquity values for ChestX-ray14 (R = -0.41, P = 7 x 10^-3^).

###

### **Supplementary Figure S4. Replication on Age Demographic**


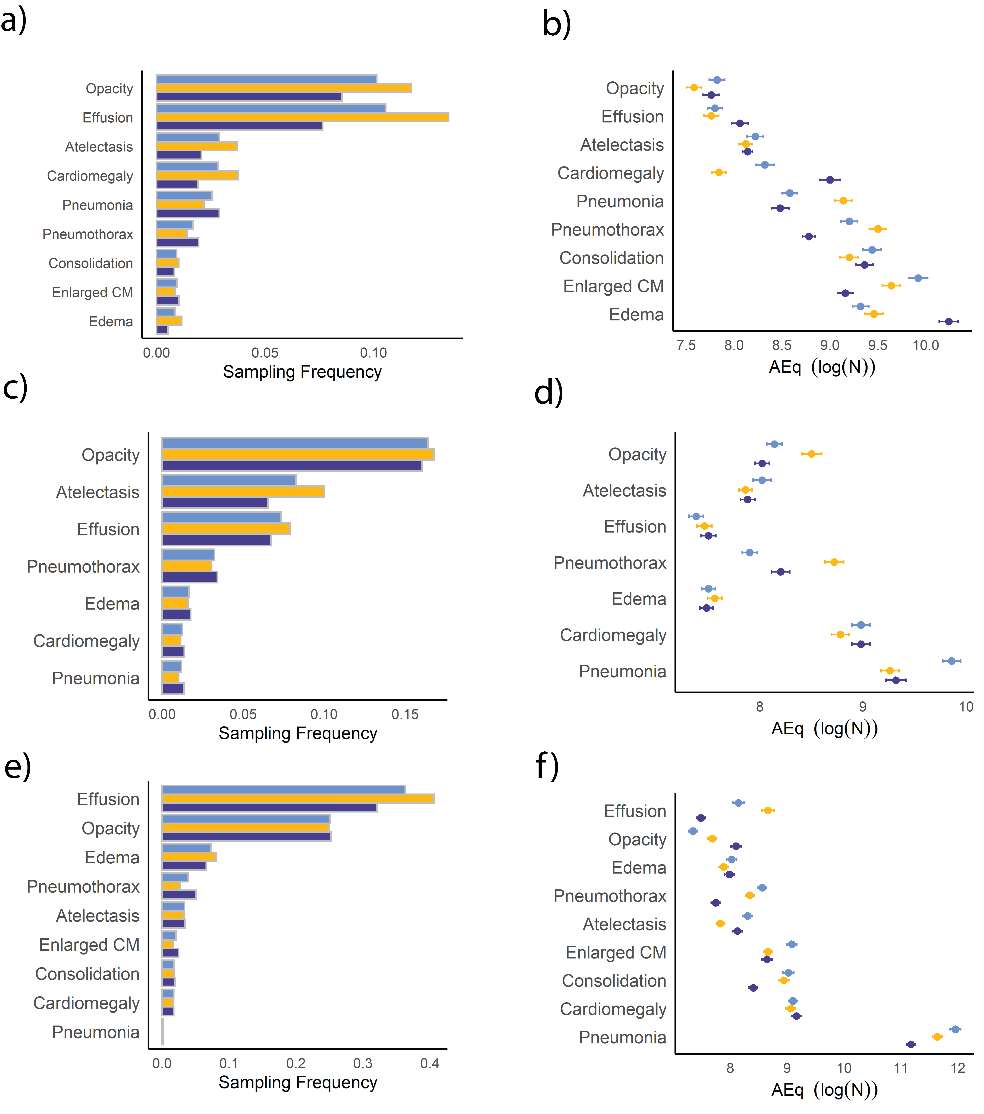


**Supplementary Figure S4**. **Replication on age demographic**. Dark blue consists of individuals age (20-40) and yellow consists of individuals from 40-60. Light blue represents the joint dataset **a)** Histogram for individuals by age on MIMIC-CXR, **b)** Corresponding AEquity values for age on MIMIC-CXR (R =-0.88, P = 2 x 10^-8^). **c)** Histogram for individuals by age for ChestX-ray14 and **d)** corresponding AEquity values (R=-0.42, P = 3.4 x 10^-5^ ). **e)** Histogram for individuals by age on CheXPert and **f)** corresponding AEquity values (R = -0.32, P = 9.2 x 10^-3^).

### **Supplementary Figure S5. AEquity Applied to Intersectional Populations.**


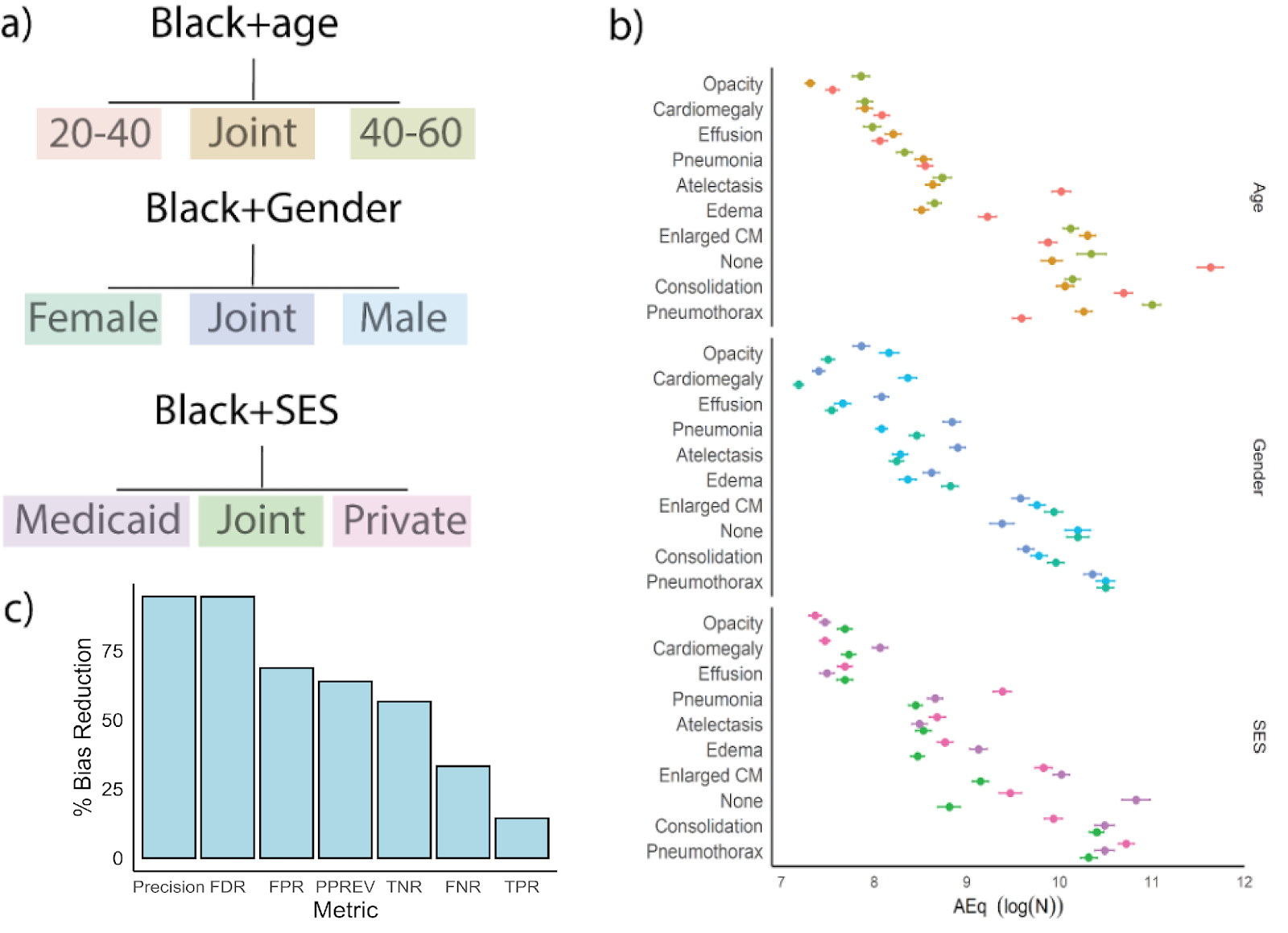


**Supplementary Figure S5**. **AEquity applied to intersectional patient populations.** (a) Age – Black patients aged 20-40 are colored red, Black patients aged 40-60 are colored green, and the combined dataset is colored gold. Sex – Male Black patients are colored lighter turquoise, Female Black patients are colored light green, and the combined dataset is colored dark blue. Socioeconomic Status – Black patients on private insurance are colored pink, Black patients on Medicaid are colored dark purple, and the combined dataset is colored dark green. (b) AEq values calculated for each group across each label. (c) Interventions applied to Black patients on Medicaid and the resulting effects on different fairness metrics.

### **Supplementary Methods**

Let there be a machine learning model *f* trained on a total of *N* samples on a dataset *X* with groups *a* and *b* and labels *h: {0, 1}*.

If X_a, 1_ is the subset *X* that belongs to group *a* and label *1*, we define AEquity as the learnability of this specific subgroup (i.e), the number of total samples of *X* required to learn a meaningful representation of the data belonging to group *a* and class *1*, where we define meaningful as the point at which increasing the number of samples yields diminishing returns on the encoded space. More formally, the AEquity value can be calculated as the following for group *a* and class *1*.

$$AEq_{a, 1}=argmin_{N}\frac{d^{2}||{f_{n}}^{-1}(f_{n}(X_{a,1}))-X_{a, 1}||_{2}}{dn^{2}}$$

A smaller n portends a better generalization performance, whereas a larger n indicates poor generalization performance when compared to other classes [^48^](https://paperpile.com/c/T74Yg1/KYTd). AEquity works by freezing the embedding layers and adding an autoencoder to the embeddings instead of traditional transfer learning where a classification block is added. We calculate equation by training an autoencoder and estimating the loss function at successively increasing sample sizes. We then plot the loss at each sample size, fit a curve, and (numerically) calculate the second derivative. We subsequently calculate the sample size at the second derivative's lowest value (arg min).

We formally define three types of interventions that AEq_i_ can support depending on the underlying cause of the bias: sampling-mediated, complexity-mediated and label-mediated interventions. Recall that bias is defined as

$$Y(x) !\perp(x \in X_{a} \cup x\in X_{b})$$

Let $\pi_{a}$ be the probability of sampling group *a*, and $\pi_{b}$ be the probability of sampling group *b*. Sampling bias is defined as when

$$\pi_{a}< \pi_{b}\Rightarrow|Q(X_{a}, Y, \hat{Y'}) - Q(X_{b}, Y, \hat{Y}')| < |Q(X_{a}, Y, \hat{Y}) - Q(X_{b}, Y, \hat{Y})|$$

Sampling bias arises when there is a low frequency of patients with a given diagnosis from a protected group. When predictive bias is driven primarily by sampling bias in the dataset, combining groups drives the AEq value at or below the over-sampled data. In sampling bias, balanced sampling from each group is sufficient to mitigate the bias because the data is equitably represented within the algorithm.

In complexity bias, a protected group presents more heterogeneously with a diagnosis, and consequently, one group exhibits a class label with greater complexity than another group. Assuming that the dataset contains all the necessary features that are fully informative for the relationship between an input feature and an output feature for all groups, the following would be true.

$$\pi_{a}=\pi_{b}\Rightarrow|Q(X_{a}, Y, \hat{Y'}) - Q(X_{b}, Y, \hat{Y}')| < |Q(X_{a}, Y, \hat{Y}) - Q(X_{b}, Y, \hat{Y})|$$

Subsequently, even if groups are drawn with equivalent frequencies, generalization performance on the more complex group is worse than other groups for the same class. For example, this could cover a group for whom the diagnostic criteria aren’t precise due to historical reasons, a group with large translational distribution shift due to geographic differences, or a group for which there are differences in data quality. In each of these three cases, the complexity of the underlying data-distribution is expected to increase.

Third, label bias occurs when outcome labels are placed incorrectly at different rates for different groups and can lead to increased misclassification errors for the affected group. For example, in published work, Black patients are more frequently labeled as requiring fewer healthcare resources despite having the same number of comorbidities because of lack of access to care .

More formally, let us assume without loss of generality that $\hat{Y(x) = 1}$ when a model predicts the outcome of interest. Let $X_{a, h}$ and $X_{b, h}$ be the subsets $X_{a}$ and $X_{b}$, respectively, consisting of patients predicted to have the outcome (like high healthcare costs). Then we define the following:

$$X_{a, h} =\{x \in X_{a}|\hat{Y(x)}= h\}$$

$$X_{b, h} =\{x \in X_{b}|\hat{Y(x)}= h\}$$

If performance-invariant bias exists, $X_{a, h}$ and $X_{b, h}$ are not drawn from the same distribution.

$$dis(X_{a, h}, X_{b, h}) > 0 \Rightarrow\hat{Y(x)} !\perp(x \in X_{a}\cup x \in X_{b})$$

where dis is a measure of distance between the distribution of independent variables within two groups with respect to a given outcome.

Several different distance metrics can be used. Simple means and variations can be computed for two normal or low-dimensional distributions.

$$dis_{a, b}(h) = E[X_{a, h}] - E[X_{b,h}]$$

$$dis_{a, b}(h) = V[X_{a, h}] - V[X_{b,h}]$$

If a distribution has a relatively high signal-to-noise ratio, then a simple Euclidean distance metric can be used.

$dis_{a, b}(h) = ||X_{a,h}-X_{b, h}||_{2}$

or in some cases, a KL-divergence can be used to approximate distance. Let *f* be a function that maps $X\Rightarrow P(Y)$

$$dis_{a, b}(h) =D_{KL}(f(X_{a, h})||f(X_{b,h}))=\sum_{x \in X} f(x_{a, h}log\frac{f(x_{a,h})}{f(x_{b, h})})$$

However, in many high-dimensional datasets, a low signal-to-noise ratio can prohibit the use of a simple Euclidean distance. We use transfer learning in high-dimensional datasets to reduce the signal-to-noise ratio and approximate the distance. We simultaneously approximate generalizability by approximating the minimum number of samples required to fine-tune the transfer learning model.

If *f* is a model trained on *X* with groups *a* and *b*, a total of *N* samples, we generate a new distance metric between groups.

$$dis_{a, b}(h):=argmin_{N}\frac{d^{2}||{f_{n}}^{-1}(f_{n}(X_{a,1}))-X_{a, 1}||_{2}}{dn^{2}}-argmin_{N}\frac{d^{2}||{f_{n}}^{-1}(f_{n}(X_{b,1}))-X_{b, 1}||_{2}}{dn^{2}}$$

$$dis_{a, b}(h):= AEq_{a, h}-AEq_{b, h}$$

We calculate this by training an autoencoder and estimating the loss function at each sample size. Next, we generated a fit a spline function on the loss versus the sample size and calculated the second derivative. Subsequently, we calculated the sample size at the lowest value (arg min) of the second derivative.

In the following section, we identify why we think it works. For clarity, we use “groups” to refer to various subpopulations, for example by race, gender, or insurance status. We will use “outcome” to refer to diagnostic findings, for example “pneumonia” and “edema”. A "protected group" refers to categories of people who are legally protected from discrimination under US law. It includes, among other things, age, sex, and race. Following an accepted framework [^1^](https://paperpile.com/c/T74Yg1/S7qq), we characterize dataset biases into main types: sampling bias; complexity bias; and label bias. Sampling bias arises when there is a low frequency of patients with a given diagnosis from a protected group. In complexity bias, a protected group presents more heterogeneously with a diagnosis, and consequently, one group exhibits a class label with greater complexity than another group. Subsequently, even if groups are drawn with equivalent frequencies, generalization performance on the more complex group is worse than other groups for the same class. For example, this could cover a group for whom the diagnostic criteria aren’t precise due to historical reasons, a group with large translational distribution shift due to geographic differences, or a group for which there are differences in data quality. In each of these three cases, the complexity of the underlying data-distribution is expected to increase. Third, label bias occurs when outcome labels are placed incorrectly at different rates for different groups and can lead to increased misclassification errors for the affected group. For example, in published work, Black patients are more frequently labeled as requiring fewer healthcare resources despite having the same number of comorbidities because of lack of access to care.

When predictive bias is driven primarily by sampling bias in the dataset, combining groups drives the AEq value at or below the over-sampled data. In sampling bias, balanced sampling from each group is sufficient to mitigate the bias because the data is equitably represented within the algorithm. When complexity bias was the only type of dataset bias, combining the groups results either in an increase in the value of AEq or an AEq closer to value that had been higher prior to the combination. In complexity bias, collecting data exclusively from the protected population, due to its relative heterogeneity compared to the over-represented group, is necessary to mitigate bias. If the algorithm exhibits residual unfairness, then the AEq will be different for two groups for the same outcome because the two groups with the same diagnostic finding are represented differently. Thus, AEquity can help quantify biases within outcome metrics and select appropriate outcomes to minimize AEq values between groups.
